# Morbid Appearances in Death by Cold

**Published:** 1861-01

**Authors:** Francis Ogston

**Affiliations:** Aberdeen


### ON THE MORBID APPEARANCES IN DEATH BY COLD.

BY FRANCIS OGSTON, M.D., OF ABERDEEN.

(1) A woman, forty-four years of age, a vagrant, was found dead by the roadside, on the morning of April 24th, 1860. She had been drinking whisky when last seen, the evening before; the night had been stormy, and there was snow on the neighboring high grounds at the time.

The body "was lying on its right side, the clothes wet, the arms by the sides, the knees approximated, the left knee fully, the right knee partly bent, the eyelids closed, the pupils natural, the mouth half open, the expression of the countenance placid, and the face, the outside of the left arm, and the fronts of both knees of a bright red hue, contrasting with the pallor of the rest of the surface, the back included, which everywhere showed well-marked cutis anserina.

"The appearances at the inspection which followed were as under: Scalp bloody; arachnoid membrane thickened and opaque; cerebral sinuses empty; blood in moderate quantity in the veins on the surface of the brain; cerebral mass paler, and containing less blood than usual; the cavities on each side of the heart, and the arteries and veins opening into them, distended to an unusal extent with blood, mostly in a fluid state, but mixed with a few red and colorless (fibrinous) clots. The blood in the heart and elsewhere nearly of the hue of arterial blood, and, unless when in bulk, after a few minutes' exposure to the air assuming wholly that appearance.

Frothy mucus in the larynx and trachea; mucous coat of the trachea minutely injected; clusters of small petechiæ on the interior of the stomach, whose contents (milk curd) exhaled a spirituous odor; liver a good deal congested; the remaining viscera of the abdomen remarkably pale and bloodless; urinary bladder enormously distended."

(2) A man aged thirty, a street porter, traveling on foot on a very cold night, drank some whisky and ale at a tavern; he then got on a cart to sleep while riding, and when the party stopped, having gone eight miles, he was found to be dead.

Dr. Ogston saw the body "in its original position on its back in the cart, with blood in moderate quantities in the nostrils, and on the face, hands, and clothes;" (he had been struck by one of his companions on the way;) "the joints rigid, the arms by the sides, and the knees half bent. Dusky-red patches were observed on the forehead, right side of the face, the chin, the outside of the right and inside of the left thighs, and on the insteps of both feet; with these exceptions, the front of the body, including the lips, markedly pale; dependent parts of the trunk and limbs livid.

"At the subsequent inspection, the following appearances presented themselves: Scalp pale; arachnoid membrane thickened and opaque; clear serum, rather abundantly under the arachnoid, in the cerebral ventricles, and at the base of the skull; excepting some fullness of the superior longitudinal sinus, the cerebral veins and sinuses contained but little blood; the mass of the encephalon unusually pale and bloodless; reddish frothy mucus in the treachea, and in considerable quantity in the bronchi; distinct œdema of the left lung; fluid blood in large quantities in the cavities on both sides of the heart, and in the arteries and veins connected with them; and the blood here as elsewhere, as in the previous case, presenting, except when in mass, a rich crimson arterial hue; the contents of the abdomen unusually pale and bloodless; a quantity of brownish fluid, smelling of beer, in the stomach; bladder enormously distended with clear urine."

Dr. Ogston remarks on the analogies observable between these cases and four others, published by him in the *British and Foreign Medico-Chirurgical Review* for October, 1855; but he very justly says that further observations are required to enable us to assume that like appearances are necessarily to be found in every case of death by cold, even in its least complicated form. The subject is not without interest in a pathological point of view, although it is chiefly important in connection with legal medicine.—*North Am. Review.*

